# The real-life heart failure patient: importance of guideline-directed medical therapy

**DOI:** 10.1007/s12471-021-01596-1

**Published:** 2021-06-17

**Authors:** G. P. J. van Hout, M. G. van der Meer

**Affiliations:** Department of Cardiology, Division Heart and Lungs, University Medical Centre Utrecht, Utrecht, The Netherlands

In the past decade the treatment of patients with heart failure with reduced ejection fraction has quickly gained momentum. Numerous randomised controlled trials (RCTs) have proved the benefit of angiotensin receptor-neprilysin inhibitors (ARNIs) and sodium-glucose transport protein 2 inhibitors (SGLT2i) in addition to the current ‘guideline-directed medical therapy’ (GDMT) [1–3].

The novel European Society of Cardiology (ESC) guideline on heart failure (HF) that will be published this year will undoubtedly recommend a substantial change in GDMT, presumably in accordance with the previously published ESC position papers and the American College of Cardiology consensus document [4, 5]. Until then, however, we will have to wait to learn what the exact role of SGLT2i and ARNIs in this new therapeutic algorithm will be.

Despite the significant improvement in pharmacotherapy for HF, previous studies have demonstrated that real-life patients do not reach the target doses that lead to the beneficial results observed in RCTs, on which the guidelines base their recommendation [6, 7]. It is therefore pivotal that the effect of GDMT in a real-life setting is determined, as done by Nauta et al. [8] in this issue of the *Netherlands Heart Journal*.

The authors retrospectively analysed the effectiveness of GDMT in terms of improvement of left ventricular function and prognosis after a mean follow-up of 3.3 years in a cohort of 378 HF patients with reduced left ventricular ejection fraction (LVEF) between 2012 and 2018. The maximal tolerated dose of each drug type was also recorded, including the reason for intolerance. All patients were newly diagnosed with HF and GDMT was initiated and/or up-titrated according to the ESC guideline [9], in an outpatient clinic under direct supervision of a specialised HF nurse, making the setting compatible with general Dutch hospitals.

The mean age of the patients was 65.5 years and just under 35% were women. After up-titration 85% of patients used a beta-blocker, 81% a renin-angiotensin-system (RAS) inhibitor and only 53% a mineralocorticoid receptor antagonist (MRA). Of the patients on medication 73% reached > 50% of the target dose of beta-blockers, for RAS inhibitors 73% and for MRA 77%. The most important reasons reported for not reaching the target dose were bradycardia, hypotension and renal dysfunction.

After an arbitrarily chosen 9‑month follow-up period, LVEF on average improved from 28% to 39%. In patients with a non-ischaemic cardiomyopathy, higher plasma levels of N‑terminal pro-B-type natriuretic peptide or older age, the LVEF was more likely to improve. The 1‑year mortality rate was 10% and a 33% mortality rate was observed after a median follow-up of 3.3 years. Each 5% increase in LVEF was associated with a hazard ratio of 0.84 (0.75–0.94, *p* = 0.002) for mortality and 0.85 (0.78–0.94, *p* = 0.001) for the combined endpoint of mortality and/or HF hospitalisation.

This study has some limitations, which should be taken into account. Importantly this is not a prospective study but rather a descriptive retrospective analysis. Moreover it is unknown if, and how long, patients were on optimal GDMT when the echocardiogram was performed at the arbitrarily chosen 9‑month follow-up assessment. It could therefore be that the improvement in the LVEF is underestimated in certain patients. Additionally, the effect of medical therapy versus device-related therapy (e.g. cardiac resynchronisation therapy) or revascularisation remains unclear in this cohort. Potential survivorship bias exists, since 35 patients died before the actual follow-up at 9 months and these patients were not included in the overall analysis. Lastly, the change in functional (New York Heart Association, NYHA) class was not described. It was mentioned that the most important reason for not reaching the recommended dose of MRA was that there was no indication at the time of the follow-up visit because patients’ functional status had improved to NYHA functional class I. However, no percentages are provided.

Despite these limitations, the article by Nauta et al. gives the reader a clear overview of the up-titration of GDMT, LVEF improvement and prognosis of real-life HF patients, albeit the relatively young age and the fact that the treatment is performed in a tertiary care centre suggest that the results could differ from an outpatient population in secondary care. The heart failure registry of the *Nederlandse Hart Registratie* (NHR) will provide more insight into this matter in the future.

Ultimately this study underlines that it is essential to verify the feasibility of implementing findings from large RCTs in daily clinical care. It shows that the majority of real-life HF patients are able to tolerate GDMT and, more importantly, seem to benefit from this regimen.
